# Cell-scale hemolysis evaluation of intervenient ventricular assist device based on dissipative particle dynamics

**DOI:** 10.3389/fphys.2023.1181423

**Published:** 2023-07-05

**Authors:** Zhike Xu, Chenghan Chen, Pengfei Hao, Feng He, Xiwen Zhang

**Affiliations:** ^1^ Applied Mechanics Laboratory, Department of Engineering Mechanics, Tsinghua University, Beijing, China; ^2^ School of Materials Science and Engineering, AVIC Aerodynamics Research Institute Joint Research Center for Advanced Materials and Anti-Icing, Tsinghua University, Beijing, China

**Keywords:** ventricular assist devices, cell-scale hemolysis simulation, dissipative particle dynamics, erythrocyte damage model, erythrocyte motion form

## Abstract

Most of the existing hemolysis mechanism studies are carried out on the macro flow scale. They assume that the erythrocyte membranes with different loads will suffer the same damage, which obviously has limitations. Thus, exploring the hemolysis mechanism through the macroscopic flow field information is a tough challenge. In order to further understand the non-physiological shear hemolysis phenomenon at the cell scale, this study used the coarse-grained erythrocytes damage model at the mesoscopic scale based on the transport dissipative particle dynamics (tDPD) method. Combined with computational fluid dynamics the hemolysis of scalarized shear stress ( τ ) in the clearance of “Impella 5.0” was evaluated under the Lagrange perspective and Euler perspective. The results from the Lagrange perspective showed that the change rate of scaled shear stress ( $\dot{\tau }$ ) was the most critical factor in damaging RBCs in the rotor region of “Impella 5.0”and other transvalvular micro-axial blood pumps. Then, we propose a dimensionless number $D_{k}$ with time integration based on $\dot{\tau }$ to evaluate hemolysis. The Dissipative particle dynamics simulation results are consistent with the $D_{k}$ evaluation results, so $\dot{\tau }$ may be an important factor in the hemolysis of VADs. Finally, we tested the hemolysis of 30% hematocrit whole blood in the “Impella 5.0” shroud clearance from the Euler perspective. Relevant results indicate that because of the wall effect, the RBCs near the impeller side are more prone to damage, and most of the cytoplasm is also gathered at the rotor side.

## 1 Introduction

Heart failure (HF) is a complex clinical syndrome caused by structural and functional damage to the filling or ejection of blood from the ventricles. According to statistics, the prevalence of HF with clinical symptoms is 1.3%–1.8%, the prevalence of asymptomatic HF is 1.5%–2%, and the prevalence of HF in people over 65 years old is as high as 10% (Basil et al., 2017). Cardiogenic shock (CS), which occurs in the late stages of HF, is very lethal, and patients must receive timely cardiac perfusion therapy to save their lives. Thoracotomy implantation of an artificial heart can be very dangerous for patients with end-stage HF. The “Impella” series transvalvular axial blood pump can be implanted from the patient’s femoral artery (similar to the implantation of a vascular stent) to provide additional flow to the heart ejection, thereby extending the patient’s life. Currently, transvalvular micro-axial blood pumps can provide cardiac perfusion at various flow rates in a minimally invasive manner. The “Impella” pump series (Abiomed, Danvers Massachusetts, US), which represents this category, is exclusive to “Abiomed” company in the United States and is the world’s largest supplier of such left ventricular assist devices (LVADs). Available clinical data showed that the “Impella” series could achieve complete left ventricular support in a short period, providing up to 5.5 L/min of flow with a good prognosis (Mehra et al., 2019). In FDA testing, the mean time to implantation of Impella 5.0 was 16.3 ± 4.7 days, 9/22 patients had a ventricular recovery, 6/22 patients were free from the risk of acute heart failure, and the 30-day survival rate was 62% (Pahuja et al., 2021). However, various blood-contacting medical devices (BCMDs), including “Impella 5.0,” produce non-physiological shear stress that can damage red blood cells (RBCs) and the freeing of large amounts of hemoglobin (Hb) into the plasma. It can cause damage to the liver and kidneys, leading to organ failure (Olsen, 2000). Several studies have shown that most of the dangerous effects caused by BCMDs are associated with blood damage (Ellis et al., 1998; Maraj et al., 1998). During the design phase of VADs, the hydraulic properties must be carefully weighed against the blood compatibility. The empirical power-law index of hemolysis (IH) formulation is the most common method when using computational simulation techniques to assess hemolysis in VADs. The researchers first use computational fluid dynamics (CFD) to evaluate various flow field information in the vicinity of VADs and then process the flow field results to predict the IH. Eq. 1 is an empirical IH formula obtained through *in vitro* experiments summarized by Giersiepen et al. (Giersiepen et al., 1990)

$$\begin{array}{c} IH=\frac{∆Hb}{Hb}=Ct^{\alpha }\tau^{\beta } \end{array}$$ (1)

where Hb is the total amount hemoglobin, ∆Hb is the amount of variation of hemoglobin in plasma. C,α,β are empirical constants, t is the exposure time of blood in a non-physiological flow field, and τ is the equivalent shear stress of the flow field. It can be found that the empirical power-law model greatly simplifies the hemolysis process, and the factors affecting hemolysis are exposure time and shear stress. Song et al. improved the IH power-law model by integrating along the blood flow trajectory, then summarized several cumulative exponential formulas for blood damage (Song et al., 2003; Yano et al., 2003). However, there are limitations to the use of macroscopic scale methods to assess hemolysis: 1. The intrinsic nature of hemolysis is erythrocyte damage, so assessing hemolysis requires consideration of the mechanical properties of the erythrocyte membrane and erythrocytes of different loads should not be subjected to the same incremental damage values (Grigioni et al., 2004; Grigioni et al., 2005). The clearance between the rotor and shroud is usually the location where VADs’ hemolysis is most severe. The link between erythrocyte kinetics and blood rheology in small-size flow becomes important (Lanotte et al., 2016).

Dissipative particle dynamics (DPD) has shown great potential for development in various simulations at the cellular scale. Fedosov et al. have developed a spring network erythrocyte model and “erythrocyte-erythrocyte” and “erythrocyte-platelet” aggregation force models based on DPD. Their study explains the Rouleaux stacking of erythrocytes and the Fahraeus-Lindquist effect of blood (Fedosov et al., 2010; Fedosov et al., 2011). Ye et al. have developed a cellular-scale model of thrombogenesis. They explored the adsorption properties of platelets (PLTs) and the process of thrombus formation in stenotic vessels using smooth dissipative particle dynamics (SDPD) (Ye et al., 2020). We have established an erythrocyte damage model and a transport dissipative particle dynamics (tDPD) based hemoglobin spillover model based on a coarse-grained erythrocyte model (Xu et al., 2022; Xu et al., 2023).

This study combined CFD and tDPD to explore the sensitive factors that lead to blood damage in non-physiological shear stress flow around “Impella 5.0". It is expected that, the cell-scale hemolysis analysis of “Impella 5.0” may hopefully guide the optimization design of VADs in the future.

The “Method” section introduces the transport dissipative particle dynamics (tDPD) and the RBC damage model in DPD systems. This section also provides the aggregation model of the RBCs population and the mapping relationship between DPD units.

In the “Result” section, the scalarized shear stress in Impella 5.0 trace lines was obtained from CFD as the boundary condition for DPD simulation to evaluate the sensitive factors of RBC damage at the cellular scale.

We propose a new hemolysis index based on cell-scale, which can calculate the damage rate of RBC membrane in a trace line and reflect the hemolysis situation. Then, this study collected a large amount of trace data and reflected it in the flow field of “Impella,” in order to observe the high hemolytic regions in the flow field. Finally, we used the traditional hemolysis index to observe the hemolysis details in the “Impella” clearance region.

## 2 Methods

### 2.1 Transport dissipative particle dynamics

Dissipative particle dynamics (DPD) was developed on the basis of molecular dynamics (MD) and combined with statistical mechanics theory (Hoogerbrugge and Koelman, 1992; Groot and Rabone, 2001). In the DPD method, the flow field is described as a series of interacting particles. The DPD uses a number of discrete particles to represent the state of the flow, representing the evolution of the system by recording the position and velocity of the particles at each time step. Eq. 2 is the basic equation of DPD.

$$\begin{array}{c} \frac{dv_{i}}{dt}=\sum \frac{1}{m}\left(F_{ij}^{C}+F_{ij}^{D}+F_{ij}^{R}\right)+F_{i}^{ext} \end{array}$$ (2)

where the interaction force $F_{ij}$ between two adjacent particles i,j consists of three components, where t is time and $v_{i},F_{i}^{ext}$ are the velocity of the i−th particle and the external force on it, respectively. The conservative force $F_{ij}^{C}$ is a soft repulsive force along the relative direction between the particles, and the magnitude of the conservative force affects the intensity of the momentum exchange within the system. The dissipative force $F_{ij}^{D}$ is used to hinder the relative motion between the particles and characterizes the viscous dissipation within the system. Its magnitude is related to the particles’ relative velocity. The random force $F_{ij}^{R}$ characterizes the random momentum exchange due to the thermal fluctuation of the system (Espanol and Warren, 1995). The expressions for the three basic forces of DPD are shown in Eq. 3.

$$\begin{array}{c} \left\{\begin{array}{l} F_{ij}^{C}=a_{ij}\left(1-r_{ij}\right)\tilde{r_{ij}}\\ F_{ij}^{D}=-\gamma \omega_{D}\left(r_{ij}\right)\left(\tilde{r_{ij}}∙v_{ij}\right)\tilde{r_{ij}}\\ F_{ij}^{R}=\sigma \omega_{R}\left(r_{ij}\right)\zeta_{ij}\tilde{r_{ij}}dt^{-\frac{1}{2}} \end{array}\right. \end{array}$$ (3)

$a_{ij}$ is the conservative force coefficient, which needs to meet $a_{ij}=75k_{B}T/\rho$ to ensure that the compressibility coefficient of water is close to the real physical scale (Groot and Warren, 1997). $r_{ij}$ is the particle distance between i−th particle and j−th particle, $\tilde{r_{ij}}$ is the unit direction vector. γ and σ are parameters of dissipative force and random force, $\omega_{D}\left(r_{ij}\right)$ and $\omega_{R}\left(r_{ij}\right)$ are the distribution function. To satisfy the Boltzmann distribution and diffusion-fluctuation theorem theory of the microcanonical ensemble system, the dissipative force and random force coefficients have the following relationship:

$$\begin{array}{c} \left\{\begin{array}{l} \omega_{D}\left(r_{ij}\right)={\left[\omega_{R}\left(r_{ij}\right)\right]}^{2}={\left(1-\frac{r_{ij}}{r_{c}}\right)}^{s}\\ \gamma =\frac{\sigma^{2}}{2k_{B}T} \end{array}\right. \end{array}$$ (4)

$k_{B}$ is the Boltzmann constant, T is the temperature of the system, $r_{C}$ is the particles cutoff radius, s is a correction factor proposed by Fan et al. (Fan et al., 2006). When using the DPD method to solve fluid dynamics problems, the relationship between particle diffusion and viscous diffusion is not physical. Reducing the numerical value of s can improve this nonphysical relationship, our paper took s=0.2 .

Li et al. (2015) added the discrete diffusion equation to the basic DPD equation. They proposed the transport dissipative particle dynamics model (tDPD), which can accomplish the advection-diffusion-reaction process (ADR) in flow The DPD-based discrete diffusion equation is derived from the continuous medium diffusion equation, and the equation framework is essentially the same as the DPD basic equation, as shown in Eq. 5.

$$\begin{array}{c} \frac{dC}{dt}=D\nabla^{2}C+Q^{S}\Rightarrow \frac{dC_{i}}{dt}=\sum \left(Q_{ij}^{D}+Q_{ij}^{R}\right)+Q_{i}^{S} \end{array}$$ (5)

In the diffusion equation for a continuous medium, C is the medium concentration, D is the diffusion coefficient, and $Q^{S}$ is the source term. In the diffusion equation for the discrete phase of DPD, $C_{i}$ is the component concentration of the particle, $Q_{i}^{S}$ is the concentration source term, and $Q_{ij}$ is the concentration flux of the corresponding particle exchange, where the Fickian flux $Q_{ij}^{D}$ represents the diffusion of the medium during convection, and the random flux $Q_{ij}^{R}$ is the medium diffusion during random momentum exchange. Eq. 6 is the basic equation of concentration diffusion of tDPD, $C_{i}$ is the component concentration of the particle, $Q_{i}^{S}$ is the concentration source term, $Q_{ij}$ is the concentration flux of the corresponding particle exchange, which can be divided into Fickian flux $Q_{ij}^{D}$ and Random flux $Q_{ij}^{R}$ :

$$\begin{array}{c} \left\{\begin{array}{l} Q_{ij}^{D}=-\kappa_{ij}\omega_{D}r_{ij}\left(C_{i}-C_{j}\right)\\ Q_{ij}^{R}=\epsilon_{ij}\omega_{R}r_{ij}\zeta_{ij}∆t^{-1/2} \end{array}\right. \end{array}$$ (6)

where $\kappa_{ij},\epsilon_{ij}$ refers to Fickian diffusion flux intensity and random diffusion flux intensity. These two coefficients also have a conversion relationship, as shown in Eq. 7.

$$\begin{array}{c} \epsilon_{ij}=m_{s}^{2}\kappa_{ij}\rho \left(C_{i}+C_{j}\right) \end{array}$$ (7)

where $m_{s}$ is the molecular weight of the solute, ρ is the number density of solvent particles. Not only can tDPD represent stochastic diffusion due to thermal fluctuation phenomena, but the particle-based computational method can also save significant computational resources when dealing with ADR problems. This study will use tDPD to simulate erythrocyte dynamics in the flow around “Impella 5.0” and to evaluate the processes of erythrocyte damage and Hb leakage to plasma. We compared the simulation results with other scholars’ hemolysis experiments *in vitro* many times and empirically set the Fickian diffusion coefficient $\kappa_{ij}={4*10}^{-8}$ in the DPD system (Zhang et al., 2011).

### 2.2 Coarse-grained erythrocyte damaged model

Prior to the twenty-first century, researchers exploring hemodynamics would typically treat blood as a Newtonian fluid. With the advancement of microfluidic techniques and rheological theories, blood-related research is set to enter a new stage. Incorporating erythrocyte modeling into blood flow has become a topic of great interest in recent years. Many optical tweezers experiments have been conducted to test the deformation of erythrocyte membranes under various states of stress and to derive the material parameters of erythrocyte membranes (Zhu et al., 2020). Dao et al. developed a viscoelastic erythrocyte membrane constitutive model. They completed the erythrocyte’s center stretching simulation using the three-dimensional finite element method, and their simulation results were also in full agreement with the optical tweezer stretching experiments (Dao et al., 2003). There are hundreds of millions of RBCs in the blood, and the RBCs have large deformation and viscoelastic characteristics. Therefore, it is very difficult to construct the fluid-structure coupling problem of RBCs-blood flow based on the traditional mesh method. Therefore, in recent years, researchers have proposed various erythrocyte models to accomplish blood flow simulation at the cellular scale with fewer computational resources. A low-dimensional model of erythrocytes consisting of ten colloidal particles was proposed by Pan et al. (Pan et al., 2010). However, the erythrocyte shape differs too much from the actual one to reproduce the deformed state of erythrocytes in flow. It is exciting that the spring network-based model of coarse-grained erythrocytes proposed by Fedosov et al. is able to achieve various deformation features of erythrocytes with a fewer number of particles (Fedosov et al., 2010). In their research tests, the error between the 500-particle constructed erythrocyte model and the real optical tweezer stretching experiment is less than 1%. The energy of the spring network-based erythrocyte model can be expressed as:

$$\begin{array}{c} V_{rbc}=V_{s}+V_{b}+V_{a}+V_{v} \end{array}$$ (8)

where $V_{s},V_{b},V_{a},V_{v}$ are the surface elastic energy, bending energy, surface area conservation penalty function and volume conservation penalty function of RBCs, respectively. The erythrocyte spring network model uses the wormlike chain nonlinear spring model, and the surface elastic energy $V_{s}$ can be expressed as:

$$\begin{array}{c} V_{s}=\sum_{j=1,2\cdots N_{s}}\left(\frac{k_{B}T∙l_{m}\left(3x_{j}^{2}-2x_{j}^{3}\right)}{4p\left(1-x_{j}\right)}+\frac{k_{p}}{l_{j}}\right) \end{array}$$ (9)

where $N_{s}$ is the number of the bonds of the erythrocyte system, p is the persistence length, kp is the spring constant, $k_{B}T$ is the energy unit, $l_{j}$ is the length of the spring j , $l_{m}$ is the maximum spring extension, and $x_{j}=l_{j}/l_{m}$ . The bending strength of erythrocyte membrane can be expressed as:

$$\begin{array}{c} V_{b}=\sum_{j=1,2\cdots N_{s}}k_{b}\left(1-\cos\left(\theta_{j}-\theta_{0}\right)\right) \end{array}$$ (10)

where $k_{b}$ is the bending constant, $\theta_{j}$ is the instantaneous angle between two adjacent triangles having the common edge j , and $\theta_{0}$ is the spontaneous angle. Finally, $V_{a}$ and $V_{v}$ are penalty functions that constrain the area and volume of RBCs, which can prevent non-physical deformation:

$$\begin{array}{l} \left\{\begin{array}{c} V_{a}=\sum_{j=1,2\cdots N_{t}}\left(k_{d}\frac{{\left(A_{j}-A_{0}\right)}^{2}}{2A_{0}}\right)+k_{a}\frac{{\left(A^{rbc}-A_{0}^{tot}\right)}^{2}}{2A_{0}^{tot}}\\ V_{v}=k_{v}\frac{{\left(V^{rbc}-V_{0}^{tot}\right)}^{2}}{2V_{0}^{tot}} \end{array}\right. \end{array}$$ (11)

where $N_{t}$ is the number of triangular mesh in the erythrocyte model, $A_{0}$ is the equilibrium value of a triangle mesh area, and $k_{d}$ , $k_{a}$ and $k_{v}$ are the local area, global area and volume constraint coefficients, respectively. $A^{rbc}$ and $V^{rbc}$ are area and volume of RBCs at the present time step, $A_{0}^{tot}$ and $V_{0}^{tot}$ are area and volume in equilibrium. The RBC particle forces corresponding to the above energies are derived from the following formula:

$$\begin{array}{c} F_{i}=-\frac{\partial V\left(x_{i}\right)}{\partial r_{i}} \end{array}$$ (12)

The DPD parameters of RBCs and PLTs used in this study were referenced from Yazdani et al. (2021), as shown in Table 1.

**TABLE 1:** Model parameters of RBCs and PLTs. $N_{v}$ is the number of particles, $\mu_{s}$ is the shear modulus, the RBC’s initial area and initial volume are $A_{0}^{tot}$ , $V_{0}^{tot}$ , the bending constant is $k_{b}$ , the constraint constant of the surface area penalty function is $k_{d}+k_{a}$ , and the constant of the volume constraint penalty function is $k_{v}$ .

	$N_{v}$	$\mu_{s}$	$A_{0}^{tot}$	$V_{0}^{tot}$	$k_{b}$	$k_{d}+k_{a}$	$k_{v}$
RBC	500	100	132.87	92.45	6.30	5000	5000
PLT	48	10000	19.63	6.02	100	5000	10000

Erythrocyte destruction is based on the ultimate force between the cytoskeleton and the cell membrane tetramer. When the force between the cell membrane tetramer is greater than 24.9 pN, the erythrocyte membrane at this location is considered to have reached a state of local maximum strain (Koshiyama and Wada, 2011). In addition, when the local maximum strain region of the erythrocyte membrane exceeds 6%, the erythrocyte membrane will produce irreversible damage and generate large-scale pores (Leverett et al., 1972). Then cytoplasm such as Hb will diffuse from the pores into the plasma. After erythrocyte damage, cytoplasmic begins to spill out of the cell membrane, and the volume of erythrocytes is no longer conserved ( $V_{v}=0$ ). Li et al. studied that the binding energy between the spectrin and cytoskeleton would decrease by 9 orders of magnitude when the intramembrane protein “Band3” embedded in the phospholipid bilayer and the anchor protein “Band4.1″connected to the spectrin and cytoskeleton were separated (Li et al., 2007). Therefore, when the cell membrane produces pores, the cytoskeleton at the corresponding location would also be fractured and relaxed, the surface elastic energy of the cell membrane becomes lower ( $V_{s}\to V_{s}^{\prime }$ ). Therefore, we made the following assumptions in the RBC damage model:

1. If the spring force in the RBC system is higher than 24.9 pN , the corresponding particles will be marked as “local maximum strain region particles”.
2. When the local maximum strain region of the erythrocyte membrane exceeds 6%, the corresponding area will produce large-scale holes, the cytoplasm will overflow the cell membrane, and the erythrocyte volume will no longer be conserved $\left(V_{v}=0\right)$ .
3. When the erythrocyte membrane is destabilized and damaged, the spectrin skeleton with immense stress begins to disconnect, manifested as connecting bonds relaxation in the coarse-grained model. Once the spring force is greater than 24.9pN, the maximum length of the spring will increase with the increase of the spring force.

The surface elastic energy provided by the spring will be changed $\left(V_{s}\to V_{s}^{\prime }\right)$ :

$$\begin{array}{l} \left\{\begin{array}{l} V_{s}^{\prime }=\sum_{j=1,2\cdots N_{s}}\left(\frac{k_{B}T∙l_{m}^{\prime }\left(3x_{j}^{\prime^{2}}-2x_{j}^{\prime^{3}}\right)}{4p\left(1-x_{j}^{\prime }\right)}+\frac{k_{p}}{l_{j}}\right)\\ l_{m}^{\prime }=l_{m}\left(r_{0}-D_{e}\left(e^{\beta \left(1-\alpha \left(\frac{F_{b}}{F_{c}}-1\right)\right)}-e^{\frac{\beta }{2}\left(1-\alpha \left(\frac{F_{b}}{F_{c}}-1\right)\right)}\right)\right) \end{array}\right. \end{array}$$ (13)

The parameters are set to $r_{0}=1.5,D_{e}=0.03,\beta =3,\alpha =20$ , $F_{b}$ is the spring force at the current moment, and $F_{c}=24.9\;pN$ is the critical force for bond relaxation. The energy composition of the damaged erythrocyte membrane $V_{rbc}^{\prime }$ can be expressed as (Xu et al., 2022):

$$\begin{array}{c} V_{rbc}^{\prime }=V_{s}^{\prime }+V_{b}+V_{a} \end{array}$$ (14)

**FIGURE 1:**
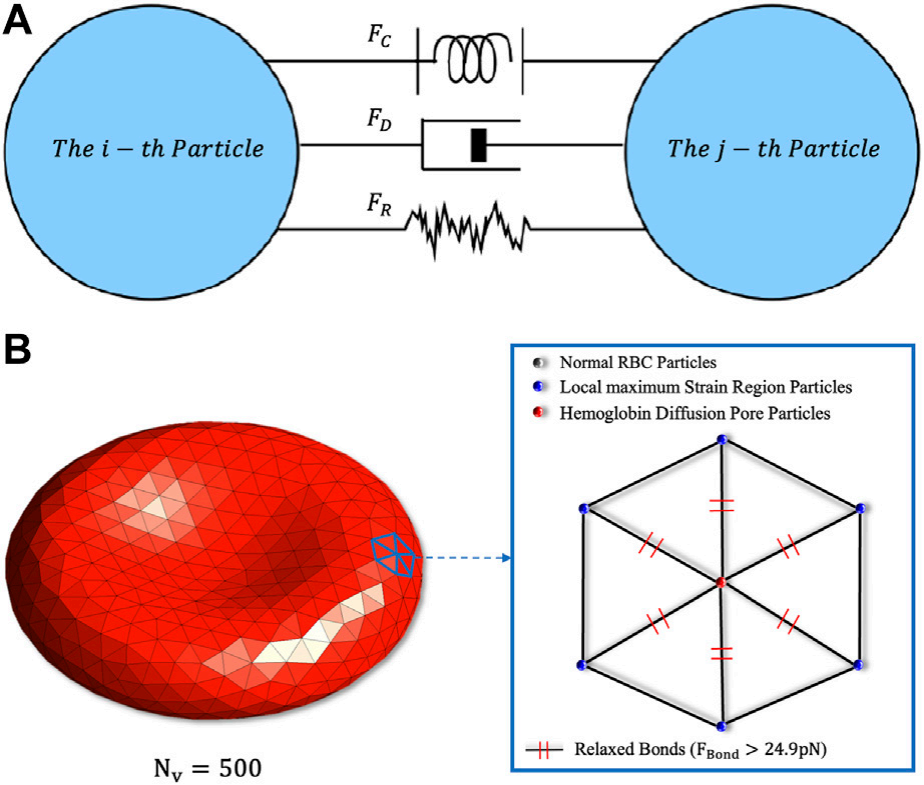
**(A)** Schematic diagram of the particles’ fundamental forces in DPD. The conservative force $F_{C}$ is a soft repulsive force indicating the intensity of the momentum exchange of the system, the dissipative force $F_{D}$ can reflect the viscosity of the system, and the random force $F_{R}$ can satisfy the dissipative rise and thermal fluctuation at the molecular scale. **(B)** Schematic diagram of the coarse-grained RBC damaged model. If all the springs around a particle reach the maximum value, this particle will be marked as a “Hemoglobin Diffusion Pore Particle” and diffuse cytoplasm into the adjacent plasma particles.

In the coarse-grained erythrocyte damage simulation, if a certain erythrocyte membrane particle and other adjacent particles reach the local maximum strain, this particle will be labeled as the “Hemoglobin Diffusion Pore Particle”, implying that the cytoskeleton within this region will undergo relaxation. Meanwhile, the pore particle will generate a source term $Q_{i}^{S}$ to diffuse Hb into the plasma, as shown in Figure 1B.

### 2.3 Aggregation force model and units mapping relationships

There is a significant aggregation effect of RBCs in healthy blood. The RBC population exhibits a rouleaux coin-like stack during low-speed movement. Therefore, modelling the aggregation force of red blood cells is necessary. Besides, in a non-physiological shear stress flow, the collision among erythrocytes is frequent, the aggregation force model can provide a great repulsive force over short distances (similar to LJ potential), preventing the overlap of RBC membranes. Fedosov et al. used Lennard-Jones (LJ) potential to represent the short-range repulsive force and Morse potential to represent the long-range attraction between RBCs (Fedosov et al., 2011). However, the time step of the DPD simulation is larger than MD, and the LJ potential may generate an extremely large force between two close particles during the simulation, causing the computing system to crash. Therefore, Yazdani et al. used only the Morse potential in DPD to build cell aggregation model (Yazdani and Karniadakis, 2016). Our study also uses the Morse potential to model the aggregation force between RBCs. The basic form of the Morse potential is shown in Eq. 8.

$$\begin{array}{c} V_{morse}=D_{e}\left(e^{2\beta \left(r_{0}-r\right)}-2\beta e^{\left(r-r_{0}\right)}\right) \end{array}$$ (15)

The equilibrium distance is set $r_{0}=0.3$ . $D_{e},\beta$ are Morse potential parameters. We tested the parameters using the aggregation force test proposed by Fedosov et al. When $D_{e}=1.6,\beta =1.5$ , a uniform stretch of 7 pN or a unilateral stretch of 3 pN will depolymerize two Rouleax-like stacked erythrocytes. The parameters of the aggregation force model between RBCs and PLTs are shown in Table 2.

**TABLE 2:** Model parameters for aggregation forces between RBCs and PLTs.

	$D_{e}$	β	$r_{0}$
RBC−RBC	1.6	1.5	0.3
RBC−PLT	2.0	2.0	1.0
PLT−PLT	2.0	2.0	1.0

The units used in the DPD method are not based on real scale units, and the results obtained from DPD simulation need to be mapped to real units through dimensional analysis. The study by Groot et al. shows that, when using the Verlet-Velocity integration algorithm, the DPD time steps less than 0.04 can stabilize the Boltzmann temperature (Groot and Warren, 1997). This study takes a time step size of 0.001, with each step corresponding to $2.14*10^{-7}$ s of the real-time unit.

The focus of this study is on the motion and deformation state of RBCs, so the length l , cell membrane shear modulus μ , and characteristic viscosity η are set as the basic units of the DPD calculation system (Yazdani et al., 2021). The corresponding relationships are shown in Table 3.

**TABLE 3:** Correspondence between DPD basic units and real physical units.

Parameter	DPD Unit	Physics Unit
$Length\;\left(l\right)$	$l_{m}=1$	$l_{p}=1\mu m$
$Shear\;modulus\left(\mu \right)$	$\mu_{m}=100$	$\mu_{p}=4.73\;\mu N/m$
$Viscosity\;\left(\eta \right)$	$\eta_{m}=117.6$	$\eta_{p}=0.0012Pa∙s$

The scaling relationship between the basic physical quantities and other physical quantities can be obtained according to the dimensional principle, as shown in Eq. 16.

$$\begin{array}{c} \left\{\begin{array}{c} \begin{array}{l} \frac{F_{p}}{F_{m}}=\frac{\mu_{p}}{\mu_{m}}\frac{l_{p}}{l_{m}}=4.73\;*\;10^{-2}\;\left(pN\right)\\ \frac{v_{p}}{v_{m}}=\frac{\mu_{p}}{\mu_{m}}\frac{\eta_{m}}{\eta_{p}}=4.64{\;*\;10}^{-3}\;\left(m/s\right) \end{array}\\ \frac{t_{p}}{t_{m}}=\frac{\eta_{p}}{\eta_{m}}\frac{\mu_{m}}{\mu_{p}}\frac{l_{p}}{l_{m}}=2.16{\;*\;10}^{-4}\;\left(s\right) \end{array}\right. \end{array}$$ (16)

where F,v,t,E are the force, velocity, time and energy respectively, the subscript p represents the real physical unit, and the subscript m represents the DPD unit. The DPD particle mass is set to m=1 , the energy unit is ${\left(k_{B}T\right)}_{m}=0.1$ , and the cutoff radius is set to $r_{c}=1.58$ . The calculation process involves a total of four types of particles, RBC particles, plasma particles, cytoplasmic particles and PLT particles. Table 4 shows the conserved force coefficients and dissipative force coefficients between different types of particles.

**TABLE 4:** DPD coefficients between different types of particles, where R is red blood cell particle, S is plasma particle, C is cytoplasmic particle, P is platelet particle, $a_{ij}$ is the conserved force coefficient, $\gamma_{ij}$ is the dissipative force coefficient.

Type	R	S	C	P
R	$a_{ij}=10.0,\gamma_{ij}=10.0$	$a_{ij}=5.0,\gamma_{ij}=45.0$	$a_{ij}=5.0,\gamma_{ij}=45.0$	$a_{ij}=2.5,\gamma_{ij}=10.0$
S	$a_{ij}=5.0,\gamma_{ij}=45.0$	$a_{ij}=2.5,\gamma_{ij}=20.0$	$a_{ij}=0.0,\gamma_{ij}=0.0$	$a_{ij}=5.0,\gamma_{ij}=45.0$
C	$a_{ij}=5.0,\gamma_{ij}=45.0$	$a_{ij}=0.0,\gamma_{ij}=0.0$	$a_{ij}=2.5,\gamma_{ij}=20.0$	$a_{ij}=0.0,\gamma_{ij}=0.0$
P	$a_{ij}=2.5,\gamma_{ij}=10.0$	$a_{ij}=5.0,\gamma_{ij}=45.0$	$a_{ij}=0.0,\gamma_{ij}=0.0$	$a_{ij}=2.5,\gamma_{ij}=10.0$

## 3 Result

### 3.1 Non-physiological shear stress flow field analysis for “Impella 5.0”

The two most commonly used computational methods for CFD evaluation of the hydraulic performance of VADs are the multiple reference coordinate method (MRF) and the slip mesh method (SMM). The MRF method “freezes” the rotor in a phase. At the same time, the boundary is given a rotational speed. The VAD’s rotation is reflected by the relative rotational speed of the boundary and the rotor, thus calculating the quasi-constant flow field of the rotor at a certain phase. In the SMM method, the fluid mesh in the rotor region will rotate with time, more closely resembling the true rotation state. Wu et al. found that the time step significantly impacts the SMM method’s computational accuracy, the calculation time step must be less than $10^{-5}\;s$ for the calculation results to converge (Wu et al., 2019). The “Impella 5.0” rotor in this study is constant at 30000 rpm, which can be considered as a steady-state calculation. Therefore, our study adopted the MRF method that consumes less computational resources.

**FIGURE 2:**
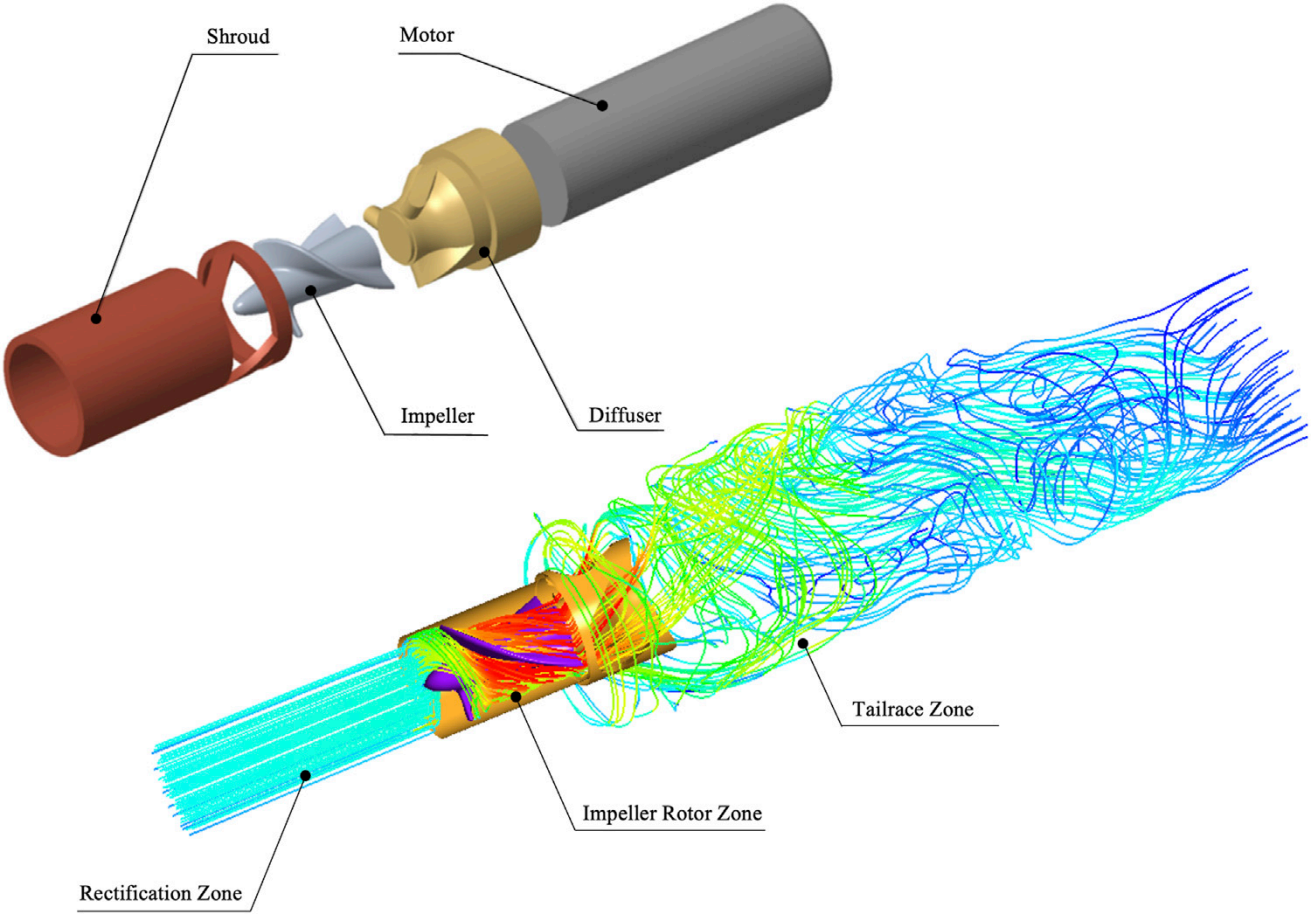
Structure and flow area of “Impella 5.0”. The “Shroud” can protect the device from working properly, the high-speed rotor “Impeller” that provides additional energy for the heart ejection. The “Diffuser” has the function of buffering and rectifying and the micro “Motor” provide energy for device. In addition, the computational area is divided into rectification zone, rotor zone and tailrace zone.

As shown in Figure 2, the “Impella 5.0” can be divided into four components. The “Shroud” is able to hold the heart valve opened and provide space for the rotor to work. The “Impeller” is the device’s rotor, which at 30,000 rpm is able to provide an additional 4L/min of ejection volume to the patient’s heart. The “Diffuser” is the device’s rear impeller, which serves as a guidance and buffering, can reduce energy loss due to vortex generation, and the “Motor” provide energy for device. This section evaluates the flow field and hydraulic performance of “Impella 5.0” using FLUENT 21.0 (ANSYS, Inc., United States). We divided the simulation area into three parts. The “Rectification Zone” enables the boundary flow to develop fully before entering the rotor area, which helps to improve the numerical simulation stability; The “Rotor Zone” is an important area for our analysis in this study. The high-speed rotor is able to boost the flow rate of 4L/min, meanwhile generating a large amount of non-physiological shear stressful flow; The “Tailrace Zone” can observe the development of tail flow and the formation of vortices. The inlet boundary condition uses a volume flow inlet boundary condition of 4L/min , the outlet boundary condition uses a pressure outlet boundary, the impeller rotation computational method uses the MRF, and the other areas are set to a no-slip wall boundary. Our CFD model uses the k−ω SST for the turbulence model, the “SIMPLE” algorithm for the mesh difference method, and no-slip wall boundary conditions. The CFD numerical calculation can be considered to have converged, when the residual of the continuous equation is less than $10^{-2}$ the residual of the k−ω equation is less than $10^{-3}$ . The average results of the last 1,000 steps are calculated as the final result.

**FIGURE 3:**
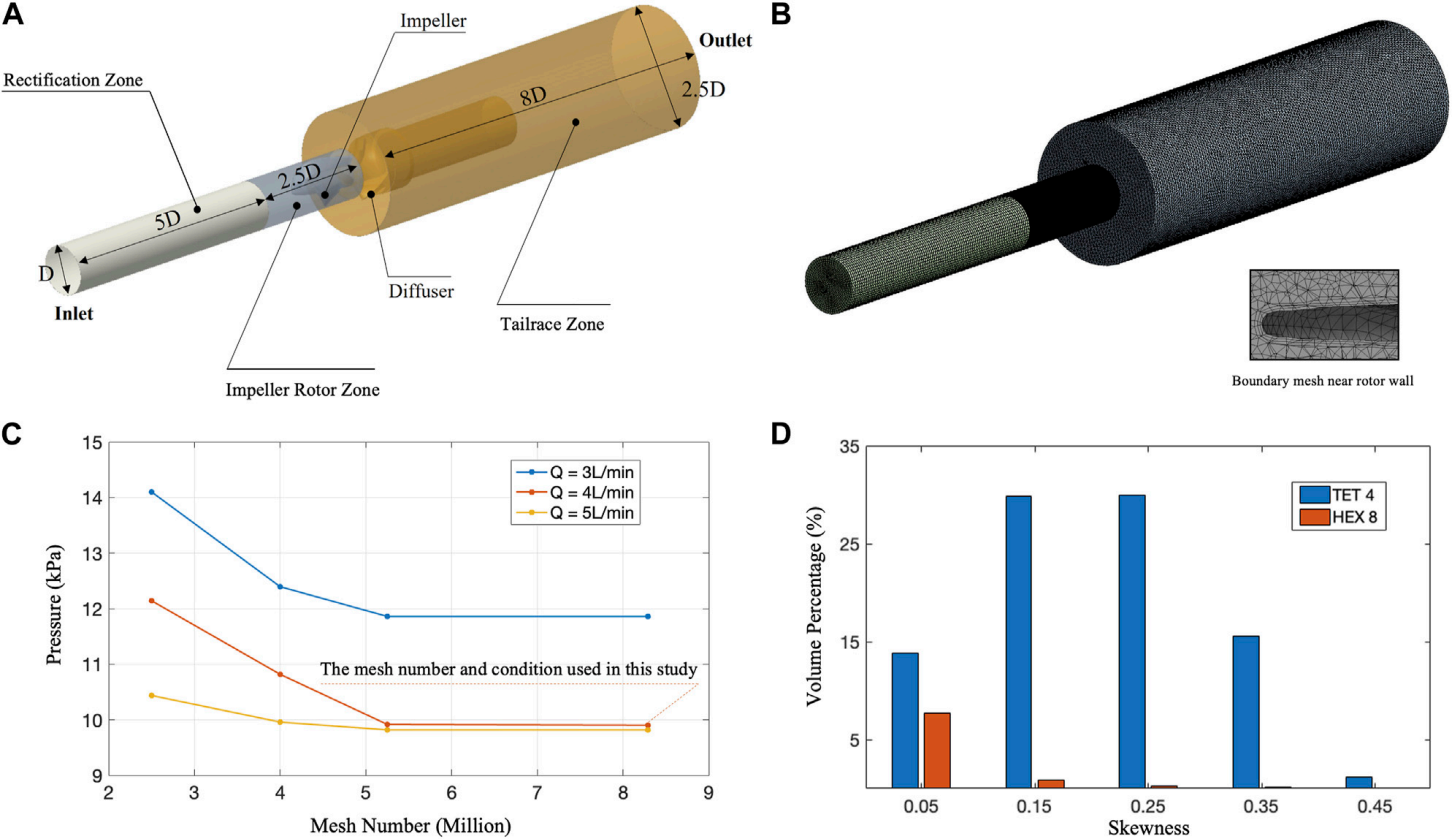
CFD mesh quality and mesh independence assessment. **(A)** Schematic diagram of CFD calculation area size $\left(D=6mm\right)$ ; **(B)** A total of 8.29 million meshes were used in the calculation domain. **(C)** Verification of mesh independence using three conditions and four mesh numbers. The horizontal axis represents the number of meshes, and the vertical axis represents the pressure difference between the inlet and outlet. **(D)** Mesh quality assessment of the 8.29 million meshes. The horizontal axis represents the skewness, and the vertical axis represents the volume percentage of different mesh quality.

The domain segmentation is shown as Figure 3A. The whole domain has a total length of 15.5D, which is 93 mm. The Mesh independence was carried out by rescaling the basic mesh in Figure 3. It can be observed that when the number of grids exceeds 5 million. The pressure change is very small, and the calculation results are independent of the number of meshes. This study used 8.29 million meshes for CFD calculations. In addition, the quality of the final meshing is shown in Figure 3D, which was judged by mesh skewness:

$$\begin{array}{c} skewness=\max\left[\frac{\theta_{\max}-\theta_{e}}{180-\theta_{e}},\frac{\theta_{e}-\theta_{\min}}{\theta_{e}}\right] \end{array}$$ (17)

where $\theta_{\max}$ , $\theta_{\min}$ are the largest and smallest angle in the face or cell, $\theta_{e}$ is the angle for an equiangular face/cell (such as 60 for a triangle, 90 for a square). Thus, the smaller the skewness is, the better the mesh is.

Scalarized the shear stress tensor not only makes it easier to quantify hemolysis, but also greatly simplifies the analysis process. Thus, tensor scalarization is usually required for the VADs hemolysis evaluation, and the most popular method of scalarization method was proposed by Bludszuweit et al. (Bludszuweit, 1995):

$$\begin{array}{c} \tau ={\left(\frac{1}{6}\sum {\left(\tau_{ii}-\tau_{jj}\right)}^{2}+\sum \tau_{ij}^{2}\right)}^{\frac{1}{2}} \end{array}$$ (18)

where τ is the scalarized shear stress and $\tau_{ij}$ is the shear stress tensor in the Cartesian coordinate system $\left(i,j=1,2,3\right)$ . The blood shear thinning model adopts the Carreau-Yasuda non-newtonian fluid model (Gijsen et al., 1999), which can be expressed as:

$$\begin{array}{c} \frac{\eta -\eta_{\infty }}{\eta_{0}-\eta_{\infty }}={\left(1+{\left(\lambda \dot{\gamma }\right)}^{a}\right)}^{\frac{n-1}{a}} \end{array}$$ (19)

η is the dynamic viscosity of blood, $\dot{\gamma }$ is the shear rate of the flow domain, other parameters are constants: $\eta_{0}=0.022Pa∙s,\eta_{\infty }=0.0022Pa∙s,\lambda =0.11s,a=0.644,n=0.392$ . Arvand et al. proved that Reynolds stress would produce a non-physical shear stress value during numerical calculations, which is usually not considered in the scalarization of shear stress (Arvand et al., 2005; Ge et al., 2008). In addition, Tullio et al. explored the effects of viscous shear stress and turbulent Reynolds stress on hemolysis of the BCMDs. They found that the scale of Reynolds stress is very large, and in micro-scale blood flow, viscous shear stress is the hemolysis dominant factor (De Tullio et al., 2009). There is no unified conclusion on how to incorporate turbulence into the process of hemolysis evaluation. Most of the serious hemolysis regions of the transvalvular micro-axial blood pumps are in the impeller clearance. The viscous stress effect on hemolysis is far stronger than Reynolds stress, so our study does not consider Reynolds stress in the VADs shroud clearance.

**FIGURE 4:**
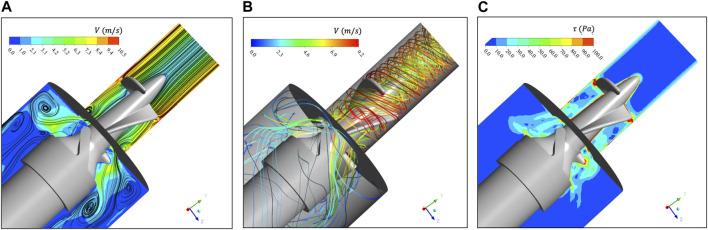
**(A)** Velocity contour and two-dimensional streamline of Y−Z section. There are two obvious eddies in the section; **(B)** Three-dimensional streamline distribution in rotor area and tailrace area. The maximum speed is at the impeller tip clearance position; **(C)** The τ contour of Y−Z section shows that the shear stress of most rotor areas is 30 Pa∼50 Pa .

Figure 4A extracts the velocity contour and two-dimensional streamlines of the Y−Z section. The flow characteristic dimension from the rotor zone to the tailrace zone is relatively large so that two vortices are formed at the corner, and these two vortices are not symmetrical. After careful observation, we can find that the tail flow field on the right side is interfered by the rear impeller, thus suppressing the formation of vortices. Figure 4B shows the three-dimensional streamline. The gap at the tip of the rotor is the position with the maximum flow velocity, the blood flow velocity can reach 10 m/s . Figure 4C shows the τ contour of Y−Z section. Areas with high shear stress $\left(\tau >100Pa\right)$ are concentrated on the gap, and the τ of most rotor areas is 30 Pa∼50 Pa .

**FIGURE 5:**
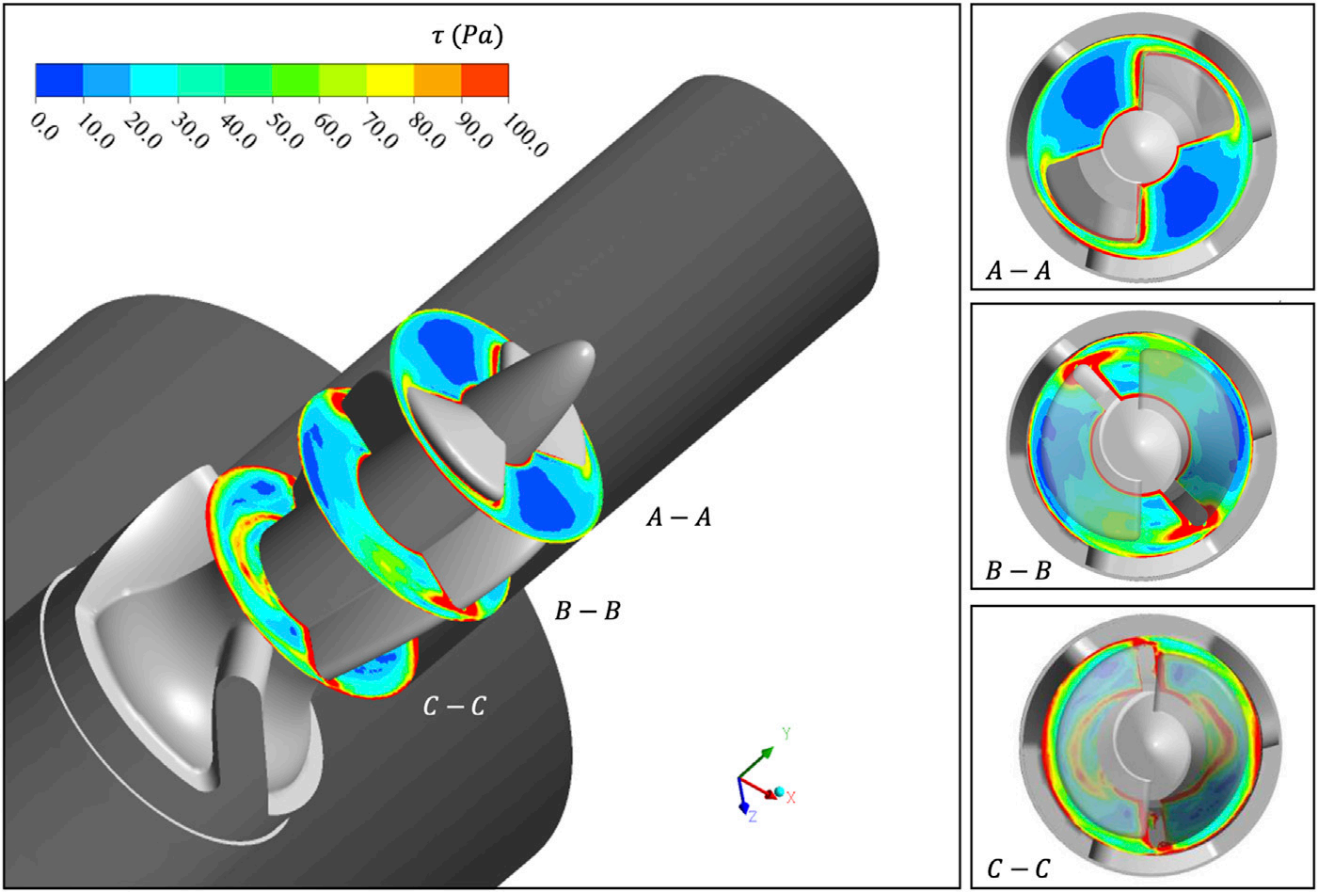
τ contour of three sections in Y direction. The velocity gradient in the clearance area between the impeller and shroud is very large and prone to hemolysis. With the development of blood flow, the area of high shear stress gradually diffused.

The τ of the blood flow is highly positively correlated with IH. We intercepted the τ data of three sections in the Y direction to facilitate a clearer analysis of the hemolytic region, as shown in Figure 5. Section A-A is the top section, and the high shear stress area is concentrated at the impeller edge. In section B-B, the high τ area of the clearance is significantly increased, and is concentrated in the clearance between the impeller and shroud. When the flow developed to the C-C position, the high stress area has already spread to most of the domain.

### 3.2 Evaluation of erythrocyte damage based on the Lagrange analysis

**FIGURE 6:**
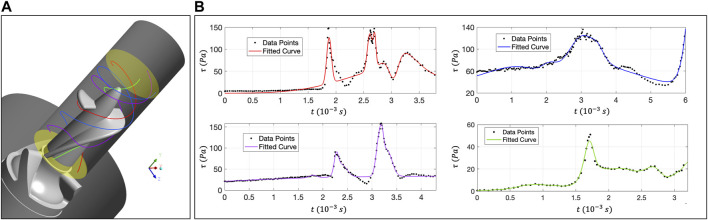
**(A)** Distribution of four trace lines in the rotor zone; **(B)** The relationship curve between τ and time on the four trace lines. The red and purple traces pass through the clearance, and the τ changes sharply. The green trace does not pass through the area prone to hemolysis, the peak τ is small.

Section 3.1 carried out a CFD-based simulation on the rotor region of “Imeplla 5.0.” We analyzed the distribution of τ in the flow field at the macro-scale to evaluate the region prone to hemolysis. In order to observe the hemolysis details of blood in the non-physiological shear stress flow from the cell-scale, we will track the movement details of RBCs in the flow field. This section will extract four characteristic trace lines from the rotor zone of “Impella 5.0,” then use the coarse-grained RBC model and DPD method to simulate the RBC motion state on the trace lines, as shown in Figure 6A. It is noteworthy that, the time step in the DPD simulation is far less than the shear stress sampling frequency on the trace line. Suppose the CFD trace data is directly imported into the DPD program. In that case, it will result in the Dirichlet velocity boundary condition being unable to derive from time, which brings more difficulties for further analysis. Therefore, we should fit a complete function curve based on the CFD data points, even if the fitting operation loses some accuracy.

The relationship curve between τ and time on the four trace lines of red, blue, purple and green is also displayed in corresponding colours in Figure 6B. The red and purple traces contact the impeller edge more frequently. The change of τ is intense, and the curve has 2-3 peaks, which can change from 10Pa to 150Pa in a short time. The degree of RBC damage may also be relatively serious. The green curve represents the flow state of most of the blood. The RBCs will enter the rear impeller along the flat position of the vane without passing through the areas prone to hemolysis, and the τ is between 20 Pa and 40 Pa.

**FIGURE 7:**
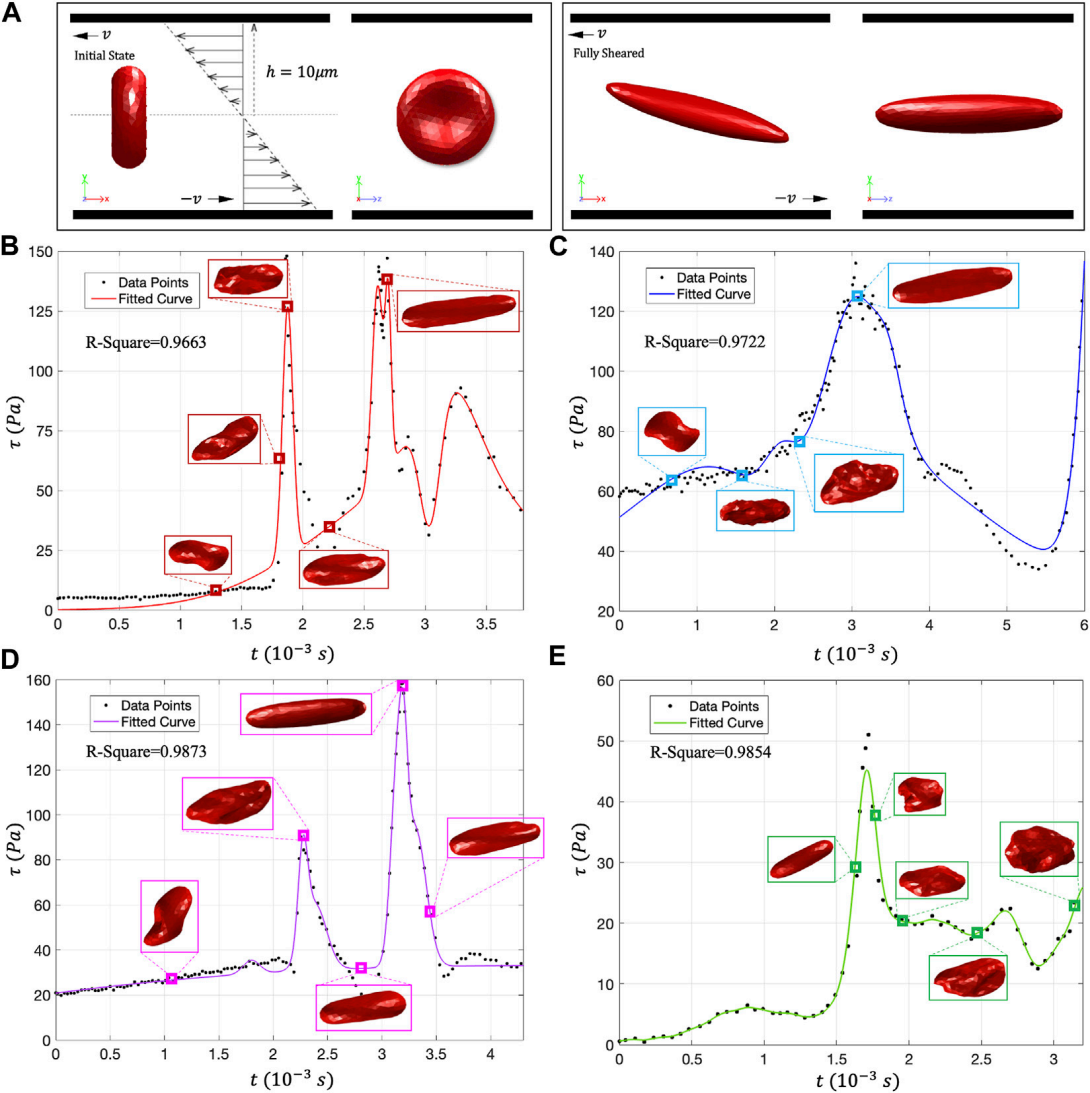
**(A)** DPD calculation setting and RBC shear flow test; **(B–E)** The movement morphology of RBCs on the four traces. Under low shear stress, the RBCs showed rolling and folding shapes. In high shear stress, almost all RBCs will evolve into tank-treading motion form and present an ellipsoidal shape.

This study uses the “Large-Scale Atomic/Molecular Massively Parallel Simulator” (LAMMPS) as the DPD particle simulation platform and uses code C++ to write specific RBC damage model functions. In DPD simulation, a 500-particle RBC model is used for simulation, and the number density of liquid particles is 3 (Feng et al., 2012). Figure 7A shows the schematic diagram of the shear test calculational settings, and the RBC’s deformation state under stable shear stress. In reality, RBCs will be subjected to multi-directional stress, and the flow characteristics are more complex. Scalarization is the most commonly used method in hemolysis analysis, so we adopted scalarized shear stress ( τ ) to reflect hemolysis and RBC’s damage. This section used the plane Couette flow formed by the movement of the boundary to characterize the τ on RBCs. To reflect the stress of micro-scale vortices on RBCs, this section evaluated the Kolmogorov scale of “Impella 5.0”, and the DPD calculation scale is also set near the Kolmogorov scale:

$$\begin{array}{c} \frac{\sigma }{d}={\left(\frac{\upsilon }{d*u}\right)}^{\frac{3}{4}}=Re^{-\frac{3}{4}} \end{array}$$ (20)

σ Is the Kolmogorov scale, the characteristic scale d=6 mm is set as the pipe diameter, $\upsilon =3.5*10^{-6}\;m^{2}/s$ is the kinematic viscosity of blood. When the flow rate Q=4L/min , u=2.2 m/s is the characteristic velocity. From Eq. 20, we can calculate σ≈12.5 μm .

Figures 7B–E shows the deformation state of RBCs under different τ . The characteristics of the red trace (Figure 7B) and the purple trace (Figure 7D) are similar, both have a great change rate of τ . The τ increases from 10 Pa to 150 Pa, taking only $10^{-4}\;s$ . Although the peak τ of these two traces can reach 160 Pa, the exposure time of RBCs in high shear stress is also relatively short. Therefore, RBC has not been fully sheared under the action of the first peak τ . With the increase of exposure time, RBC after the second peak will change into an elongated ellipsoid-shaped under the action of shear stress flow. Although the peak in the blue trace (Figure 7C) is only 120 Pa, the time of RBCs exposed to shear stress flow above 60 Pa is much longer than that in the red and purple trace lines. Therefore, under the 120Pa peak, the RBCs also change into tank-treading motion form. The peak of the green trace is relatively low. In the low shear stress, the RBC motion form presents a rolling, folding and multilobe shape, and the changing shape is relatively rich. According to the simulation results based on Lagrange method. In addition to τ and exposure time, the shear stress change rate $\dot{\tau }$ may also be one of the sensitive factors of hemolysis.

**FIGURE 8:**
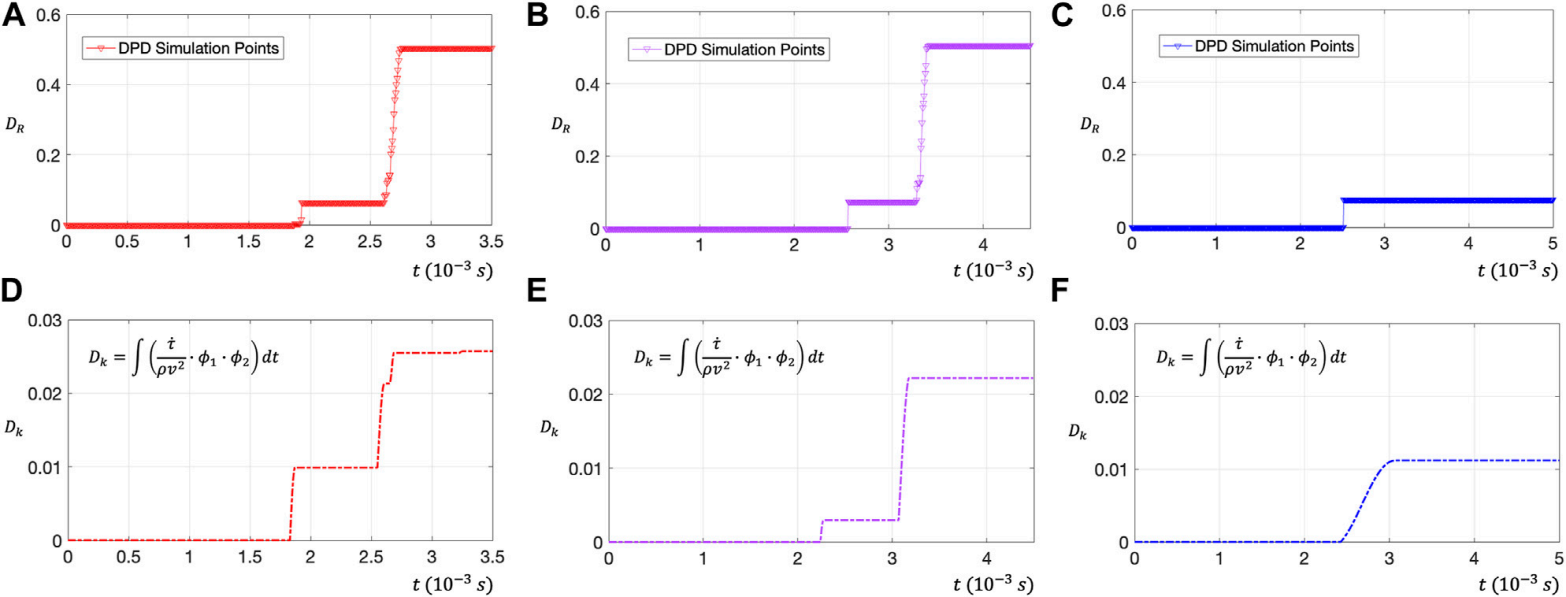
**(A–C)** Use DPD to evaluate the RBC membrane surface damage rate $D_{R}$ on red (597 data points), purple (680 data points) and blue traces (847 data points). These three curves are characterized by stepwise growth. **(D–F)** Use the time integral dimensionless number $D_{k}$ to evaluate the RBC damage amount on the three trace lines. After comparison, it can be found that, although there are some errors in the peak value, the time point of RBC injury is coincident.

To further verify the effect of shear stress change rate ( $\dot{\tau }$ ) on erythrocyte damage. In the DPD simulation, we recorded the ratio of the local maximum stress area (RBC’s damage area) to the total area of the cell membrane ( $D_{R}$ ) every 500 computing steps. So as to evaluate the damage degree of RBCs quantitatively, as shown in Figure 8A–C. The RBCs on the green trace line are not damaged, so this paragraph does not discuss the green trace. The RBC damage curve simulated by DPD has a stepwise growth trend. When the trace line passes through the high shear stress flow in the impeller clearance every time, the cytoskeleton breaks suddenly after exceeding the maximum stress, and the damage to the erythrocyte membrane will increase sharply. Interestingly, although the peak τ of the blue trace can reach 120 Pa and the exposure time is relatively long, the RBCs have been fully stretched into an ellipsoid-shaped, and the damage rate is the lowest, less than 10%. The red and purple traces have two peak regions, and the corresponding $\dot{\tau }$ is also much higher than the blue trace, and the RBC damage rate is as high as 50%. So far, in “Imeplla 5.0” and other transvalvular micro-axial blood pumps, we may judge that the $\dot{\tau }$ is one of the most important factors affecting hemolysis. Moreover, there are more regions of high $\dot{\tau }$ in the trace passing through the impeller clearance frequently, resulting in more serious hemolysis. Further, we take $\dot{\tau }$ as the main variable to construct a dimensionless number ( $D_{k}$ ) related to RBC damage, and its expression is as follows:

$$\begin{array}{c} D_{k}=\int \left(\frac{\dot{\tau }}{\rho v^{2}}∙\phi_{1}∙\phi_{2}\right)dt \end{array}$$ (21)

The dimensionless number of RBC damage $D_{k}$ should be expressed as an integral relationship with time, $\rho v^{2}$ is the average energy of blood flow in the rotor zone. Our previous studies have shown that RBCs can be damaged when the shear stress is above 80 Pa. Thus, in order to correctly express the accumulation of $\dot{\tau }$ on blood damage, we add two limiters $\phi_{1},\phi_{2}$ to the dimensionless integration formula. $\phi_{1}$ indicates that only flow acceleration can injure RBCs, while flow deceleration does not affect RBC damage; $\phi_{2}$ represents that the shear stress flow above 80 Pa can affect the damage amount of RBCs (Xu et al., 2022).

$$\begin{array}{c} \phi_{1}=\left\{\begin{array}{c} 1\;\left(\dot{\tau }>0\right)\\ 0\;\left(\dot{\tau }<0\right) \end{array}\right. \end{array}$$ (22)

$$\begin{array}{c} \phi_{2}=\left\{\begin{array}{c} 1\;\left(\tau >80\;Pa\right)\\ 0\;\left(\tau <80\;Pa\right) \end{array}\right. \end{array}$$ (23)

Figures 8D–F shows the relationship between the dimensionless number $D_{k}$ and time, which can directly evaluate the blood injury status through flow field information. Comparing the $D_{k}-t$ curve obtained from the flow field information with the $D_{R}-t$ curve obtained from the DPD simulation, although there are some errors in the damage peak data, the damage time point can coincide. This phenomenon also explains that the $\dot{\tau }$ may be an important factor leading to hemolysis in the high-speed rotating impeller zone. Thus, in designing VADs, it may be necessary to consider adding a flow buffer layer to reduce the shear stress change rate to inhibit hemolysis.

### 3.3 Hemolysis evaluation in position of rotor clearance

**FIGURE 9:**
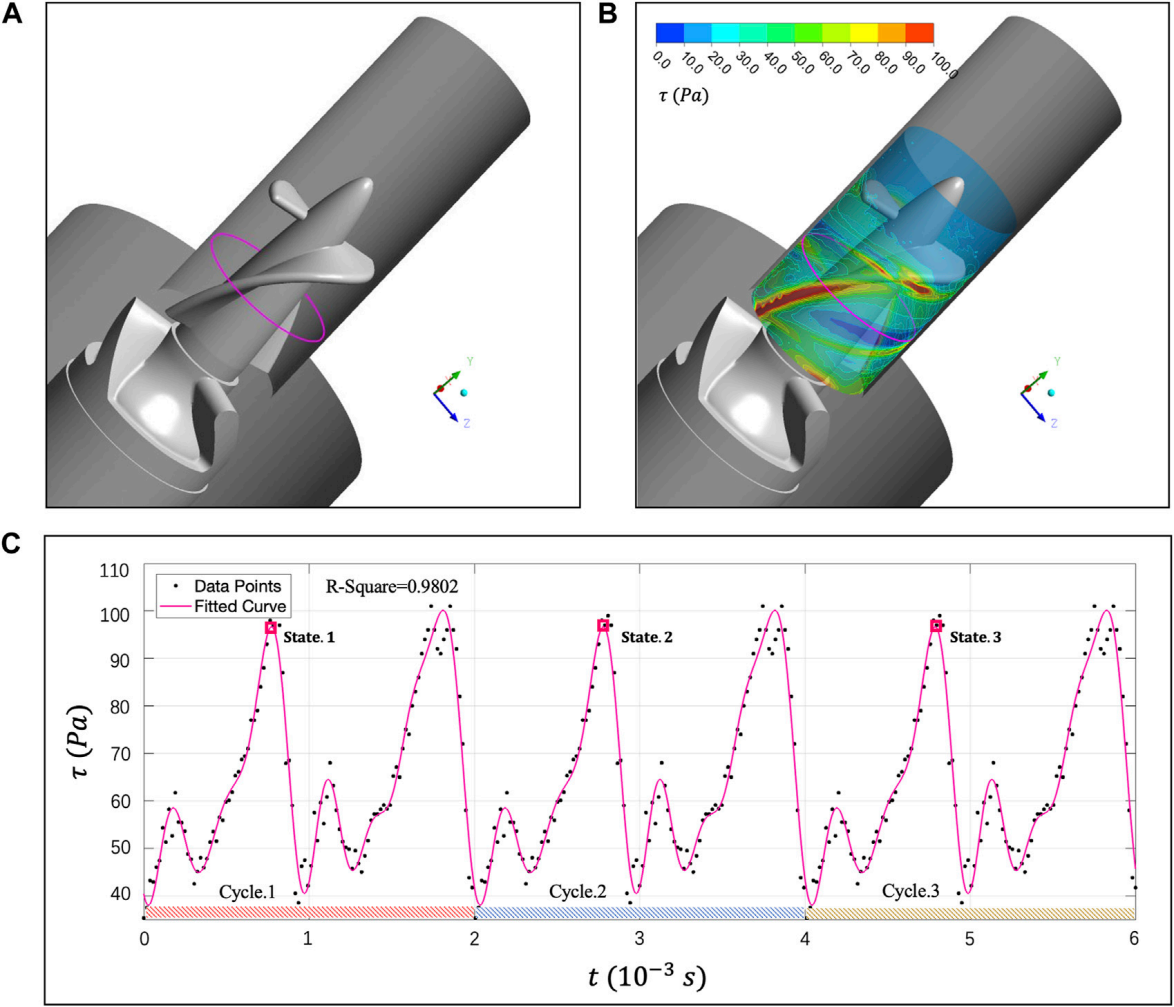
**(A)** Magenta curve represents the τ monitoring area during rotor one cycle rotation; **(B)** τ distribution contour of “Impella 5.0″clearance. The τ of the flow field in contact with the impeller is as high as 100 Pa. **(C)** Monitor the τ -t curve of the specific location within three rotation cycles of the rotor.

To analyze erythrocyte damage from Euler perspective, this section will monitor the τ of a fixed area in the gap. The monitoring track will also change into a complete circular edge when the impeller rotates for one circle. Figure 9A shows the intercepted data area and is marked as a magenta curve. At the same time, we extracted the τ distribution contour in the rotor clearance (Figure 9B). The τ at the contact position with the impeller is higher than 100 Pa, and the average τ in the clearance is between 60 Pa and 70 Pa. Figure 9C shows the τ -t curve of the monitoring area in three rotating cycles, lasting for $6*10^{-3}\;s$ .

**FIGURE 10:**
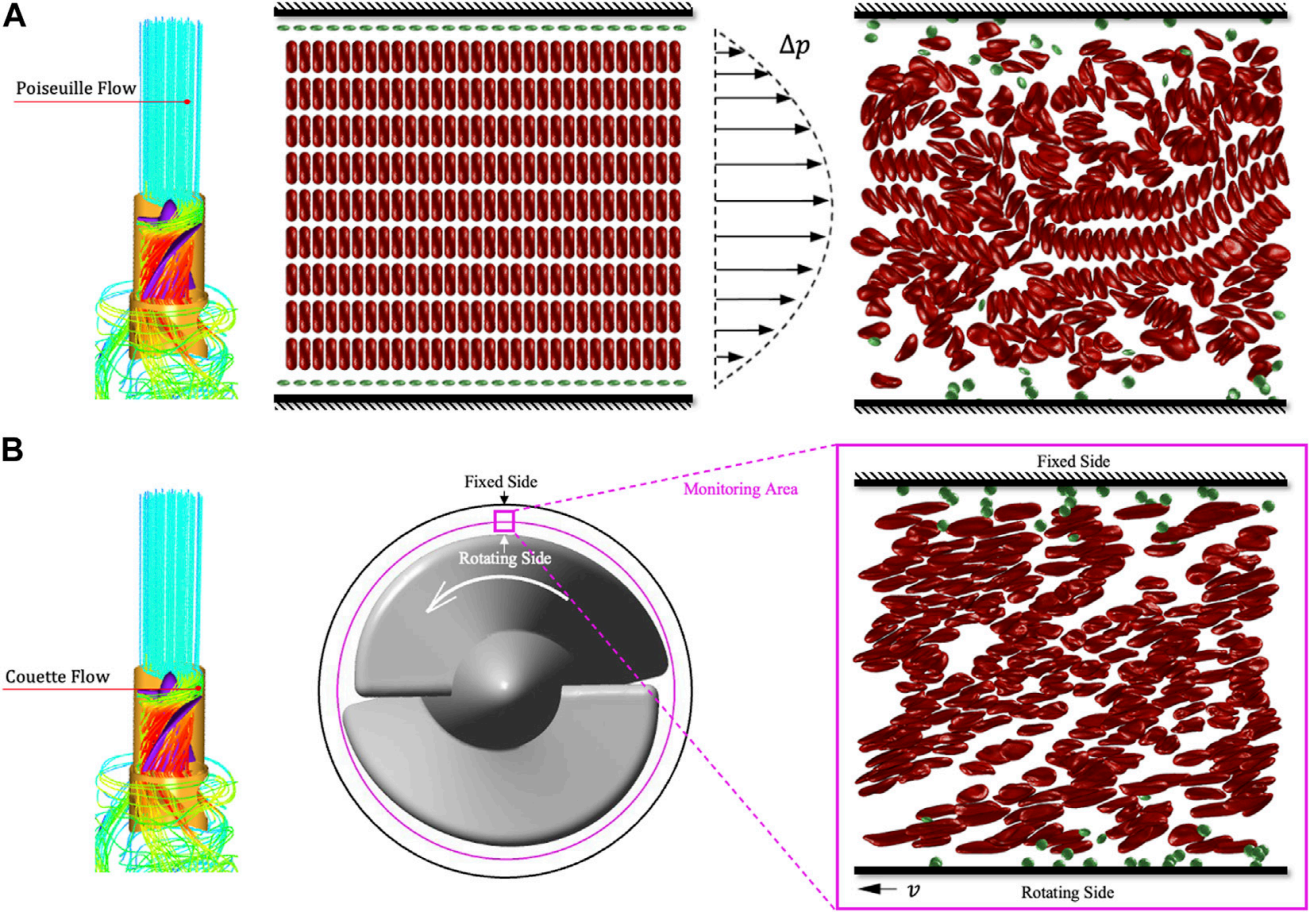
Schematic diagram of tDPD whole blood hemolysis simulation. **(A)** The whole blood flow in the rectification area is regarded as the Poiseuille flow driven by blood pressure. **(B)** The flow in the rotor area is regarded as Couette flow. Then the velocity gradient generated by the movable wall can simulate the shear stress generated by the impeller in the clearance.

Next, we will use tDPD to analyze the RBCs’ population morphology and 30% hematocrit whole blood hemolysis in the 100 μm×100 μm×5 μm area. There are 270 RBCs and 50 PLTs in the calculation area. The simulation involves 406200 particles, 410280 bonds, 273520 angles and 410280 dihedrals. It takes about 48 h to compute every 100000 calculation steps. The figure hides plasma particles and cytoplasmic particles to show the simulation results more clearly. Figure 10 shows the process of tDPD simulating whole blood hemolysis under Euler’s perspective, and the simulation are divided into two steps. When the blood has not entered the rotor area, it will be Poiseuille flow driven by blood pressure. First, we should simulate the RBC movement and random distribution in Poiseuille flow. We use a pressure gradient ∆p=80mmHg to simulate the whole blood flow in the rectification zone, as shown in Figure 10A. This simulation step lasted 400000 steps, and the movement of RBCs has become stable. It can be found that the RBCs in the centre still maintain the “Rouleux” coin-like stack, which is consistent with the findings of Fedosov et al. and Yazdani et al. (Fedosov et al., 2014; Yazdani and Karniadakis, 2016). Figure 10B shows the second step of the simulation. When blood enters the rotor area, the flow in the impeller clearance can be simplified as Couette flow. We regard the tDPD simulation area as the monitoring region in the clearance between the impeller and the shroud. Because of the tiny monitoring area, the influence of curvature on flow is ignored in the tDPD simulation process. The moving wall at the lower side of the simulation area represents the rotor side, which can generate shear stress and injure RBCs.

**FIGURE 11:**
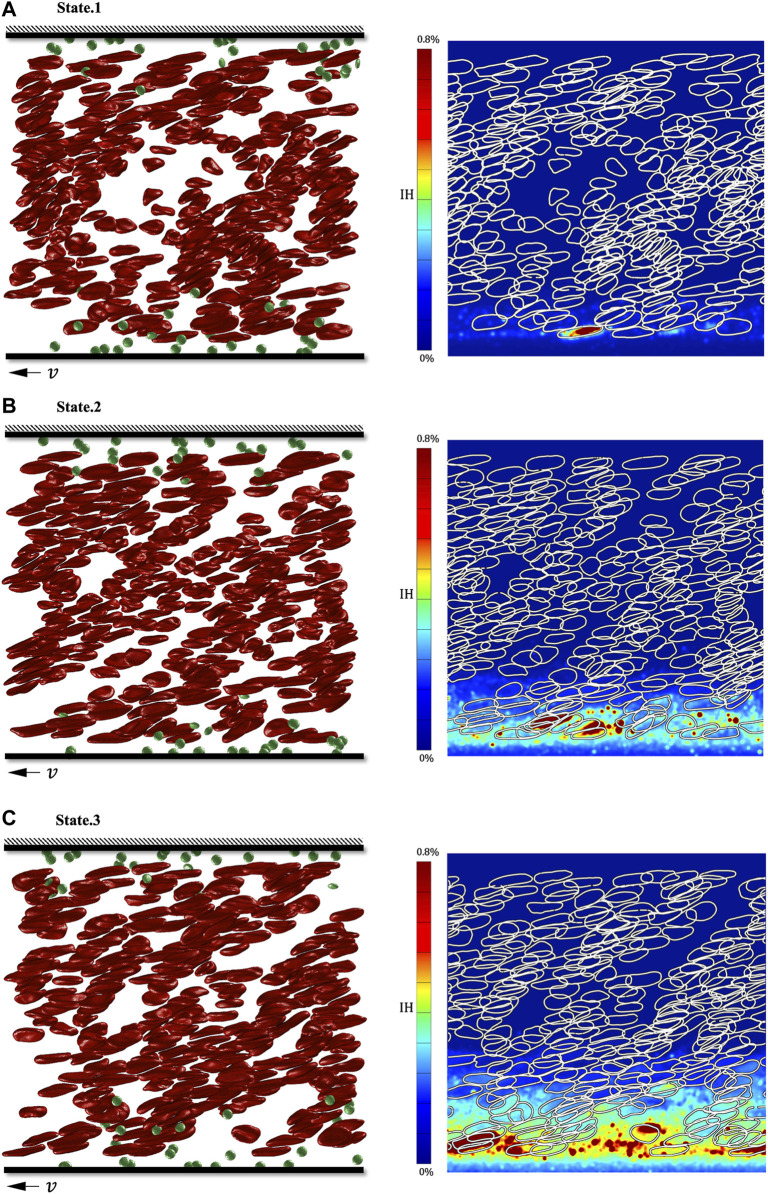
Simulation results of three shear stress peaks in the shroud clearance flow domain. **(A)** RBC’s deformation and hemoglobin distribution at 0.00082s; **(B)** RBC’s deformation and hemoglobin distribution at 0.00282s; **(C)** RBC’s deformation and hemoglobin distribution at 0.00482s.

We show the tDPD simulation results of the first, third and fifth peaks at the monitoring region, and the corresponding time points are marked in Figure 9C. IH calculation formula is as follows (Sohrabi and Liu, 2017):

$$\begin{array}{c} IH=\left(1-H_{ct}\right)\frac{Hb_{P}-Hb_{p}^{0}}{Hb_{B}}\approx \left(1-H_{ct}\right)\frac{Hb_{P}}{Hb_{B}} \end{array}$$ (24)

where $H_{ct}$ is hematocrit, $Hb_{B}$ is the total hemoglobin, $Hb_{P}$ is the hemoglobin in plasma, and $Hb_{p}^{0}$ is the hemoglobin in healthy whole blood plasma. In healthy blood, no hemoglobin is free in plasma $\left(Hb_{p}^{0}=0\right)$ . Thus, this hemolysis index formula can be simplified. As shown in Figure 11A, most RBCs are still in the aggregation state in the first peak because of the short exposure time. Due to the interface non-slip effect, the RBCs near the wall have large deformation, while a small number of RBCs on the side of the rotor have been damaged. With the increased exposure time, most RBCs have shown a tank-treading motion state and were no longer clustered. The Hb overflowing from the damaged RBCs on the rotor side and the IH value near the wall increased significantly (Figures 11B, C). When the whole blood enters the rotor zone, RBCs are more likely to be damaged on the side of the rotor. Other cytoplasms, such as Hb, are also concentrated on the side of the rotor. It is worth noting that the more Hb and damaged RBCs, PLTs, and coagulation factors would be gathered on the side of the rotor, which may also be an indirect reason for the frequent attachment of thrombosis and gel on the VADs impeller.

## 4 Discussion

### 4.1 Conclusion

Combining computational fluid dynamics (CFD) and transport dissipative particle dynamics (tDPD), this paper evaluates the hemolysis in the rotor zone of “Impella 5.0” at the macro-scale and cell-scale. Then, we analyze the damage of RBCs and whole blood from the “Lagrange perspective” and the “Euler perspective” and summarize a dimensionless number $D_{k}$ used to evaluate the blood compatibility of “Impella 5.0” and other transvalvular micro-axial blood pumps.

We first used CFD to focus on the velocity and scalarized shear stress ( τ ) distribution in the “Impella 5.0” rotor region. The velocity gradient and τ at the clearance between the impeller and the shroud are the highest, which are the areas prone to hemolysis in the rotor zone.

Then, we extracted four trace lines of the rotor region and analyzed the movement and deformation of RBCs on the four traces using the DPD method. Under low shear stress, RBCs can show various deformation states, such as rolling, folding, and multilobe. However, RBCs exposed to high shear stress for a long time will present ellipsoid-shaped, and the cell membrane will also be irreversibly damaged.

Notably, in the rotor zone of “Impella 5.0,” the change rate of scalarized shear stress $\dot{\tau }$ may be one of the most important factors for RBC damage and hemolysis. Therefore, we proposed a dimensionless number $D_{k}$ of hemolysis based on $\dot{\tau }$ in the Lagrange perspective. After the comparison, it was found that the result $D_{R}$ of RBC damage simulation was consistent with the result $D_{k}$ of dimensionless number evaluation, which can preliminarily confirm our conclusion. Therefore, in designing VADs, it may be necessary to add buffer segments to reduce the RBC’s damage of $\dot{\tau }$ to reduce hemolysis symptoms effectively.

Finally, we continuously observed the τ of the three rotation cycles in the “Imeplla 5.0”clearance region from the Euler perspective. We used tDPD to simulate the RBC motion form and IH distribution with a hematocrit of 30% of the whole blood. Due to the boundary effect, the RBCs at the rotor side boundary are easily damaged, and the Hb and other cytoplasm are concentrated on the rotor side. In the same way, damaged RBCs, PLTs, and coagulation factors would also gather on the side of the rotor, which may also be the reason why the VADs impeller often produces thrombus and gel.

### 4.2 Limitations

Nevertheless, there are still many unsolvable limitations in this study. 1. Considering that the viscous shear stress dominates the non-physiological shear stress in the clearance, this study simplifies the model and does not consider the role of turbulent Reynolds stress. 2. DPD method is a mesoscopic scale calculation method which cannot produce complex flow. Thus, we simplify the blood flow in the clearance and use the plane Couette flow to characterize and quantify the damage of shear stress to RBCs. 3. The hemolysis index proposed in this study is an integral formula of Lagrange’s perspective. In addition, $\dot{\tau }$ is also an index that is difficult to measure in the process of *in vitro* tests. The construction of the hemolysis formula based on the hemolysis principle from Euler’s perspective is still a huge challenge.

In addition, it is necessary to use tensors to characterize the damage of red blood cells. Currently, many scholars have obtained the relationship between red blood cell deformation and flow tensor (Faghih and Sharp, 2020), but no researchers have considered the mapping relationship between flow tensor and red blood cell damage. In future work, we need to construct a flow stress tensor based on complex flow in DPD method, then further improve mesoscale hemolysis research by combining flow tensor with blood damage models.

Multi-scale blood injury research is a huge challenge, as it is difficult to couple macroscopic hemolysis phenomena with microscopic damage to red blood cells. The RANS model used in this study is obviously not enough to characterize the Kolmogorov scale vortex that causes damage to red blood cells (Antiga and Steinman, 2009). Therefore, in future, we plan on combining the DNS method and the DPD method which may make the blood damage research more accurate.

## Data Availability

The raw data supporting the conclusion of this article will be made available by the authors, without undue reservation.
